# FOXA1: A Pioneer of Nuclear Receptor Action in Breast Cancer

**DOI:** 10.3390/cancers13205205

**Published:** 2021-10-17

**Authors:** Darcie D. Seachrist, Lindsey J. Anstine, Ruth A. Keri

**Affiliations:** 1 Department of Cancer Biology, Lerner Research Institute, Cleveland Clinic, Cleveland, OH 44195, USA; seachrd@ccf.org; 2 Case Comprehensive Cancer Center, Case Western Reserve University School of Medicine, Cleveland, OH 44106, USA; anstinl2@ccf.org; 3 Department of Pharmacology, Case Western Reserve University, Cleveland, OH 44106, USA; 4 Department of Cancer Biology, Cleveland Clinic Lerner College of Medicine of Case Western Reserve University, Cleveland, OH 44106, USA; 5 Department of Genetics and Genome Sciences, Case Western Reserve University, Cleveland, OH 44106, USA

**Keywords:** FOXA1, nuclear receptors, estrogen receptor, progesterone receptor, androgen receptor, glucocorticoid receptor, endocrine resistance, breast cancer

## Abstract

**Simple Summary:**

Factors such as estrogen, progesterone, and androgen receptors, also known as nuclear receptors, are abundantly expressed in the majority of breast cancers where they serve as critical regulators of tumor growth and metastatic disease. The ability of these receptors to regulate breast cancer cell functions depends on their interactions with DNA to control the activity of a large number of genes. FOXA1 is a protein that is highly expressed in a majority of breast cancers and its binding to DNA helps define which genes are regulated by nuclear receptors. This review discusses the current literature on how FOXA1 controls gene activity, cell biology, and the response of breast cancers to hormone therapies. It also offers areas of future study to identify roles of FOXA1 in controlling breast cancers that is independent of its regulation of nuclear receptors.

**Abstract:**

The pioneering function of FOXA1 establishes estrogen-responsive transcriptomes in luminal breast cancer. Dysregulated FOXA1 chromatin occupancy through focal amplification, mutation, or cofactor recruitment modulates estrogen receptor (ER) transcriptional programs and drives endocrine-resistant disease. However, ER is not the sole nuclear receptor (NR) expressed in breast cancers, nor is it the only NR for which FOXA1 serves as a licensing factor. Receptors for androgens, glucocorticoids, and progesterone are also found in the majority of breast cancers, and their functions are also impacted by FOXA1. These NRs interface with ER transcriptional programs and, depending on their activation level, can reprogram FOXA1-ER cistromes. Thus, NR interplay contributes to endocrine therapy response and resistance and may provide a vulnerability for future therapeutic benefit in patients. Herein, we review what is known regarding FOXA1 regulation of NR function in breast cancer in the context of cell identity, endocrine resistance, and NR crosstalk in breast cancer progression and treatment.

## 1. Introduction

The pioneering functions of FOXA1 and nuclear receptor (NR) transcriptional programs are tightly coupled in breast cancer. Research over the last two decades has elucidated the role of FOXA1 in regulating the ability of steroid nuclear receptors to control transcription, predominantly in hormone-responsive tissues such as the breast, and has revealed critical roles of select FOXA1/NR partnerships in both organ development and cancer progression (reviewed in [1]). Among the ~40 NR family members expressed in breast cancer [2], the receptors for estrogens, androgens, glucocorticoids, and retinoic acid receptor are particularly dependent upon FOXA1 for chromatin access and transcriptional regulation, with the estrogen receptor-α isoform (ER) being the most clinically relevant. A second isoform of estrogen receptor is also expressed in breast cancer (ER-β/ESR2); however, the relative contributions of ER-β to this disease are less well understood (reviewed in [3]). Other NRs also play critical roles in breast cancer progression, such as vitamin D receptor [4], and liver receptor homolog-1 [5]; however, these NRs function independent of FOXA1 for chromatin access. 

The expression of ER in breast cancer significantly dictates treatment course and is prognostic of patient outcomes [6,7,8]. Indeed, therapeutic targeting of ER signaling for breast cancer management using selective ER modulators (SERMs; i.e., tamoxifen), aromatase inhibitors (i.e., anastrozole), or selective ER degraders (SERDS; i.e., fulvestrant) provides long-term benefit for the majority of patients with early-stage disease and extends the lives of many patients with advanced lesions. Progesterone receptor is a direct transcriptional target of ER. PR expression is a biomarker of active ER and is associated with increased breast cancer patient survival. Together, these NRs clinically define the luminal breast cancer subtypes (luminal A and B), which account for the majority of breast malignancies (~70%). The remaining 30% of breast cancers represent either the human epidermal growth factor receptor (HER2)-enriched subtype or triple negative breast cancers (TNBC), so named as they lack ER, PR, and HER2 expression. Apart from the well-established role of estrogens and ER in breast cancer, tumor-promoting and suppressing functions of androgens and glucocorticoids is still under investigation (reviewed in [3,4]). Importantly, NR function in breast cancer is primarily dictated by cellular context and the coordinate expression of factors that control their expression and function. One of the essential factors that dictate steroid NR signaling in breast cancer is the transcription factor, FOXA1. FOXA1 remodels chromatin and acts as a licensing factor to provide access of NRs to their cognate cis-regulatory regions. Together, FOXA1 and NRs execute subtype-specific transcriptional programs that can confer differences in the course of disease and patient outcomes. In this review, we provide an update on the role of FOXA1 in controlling NR function in breast cancer and identify areas requiring additional focus to delineate the full spectrum of FOXA1 functions in this disease.

## 2. FOXA1 Family History

Over 30 years ago, Darnell and colleagues sought to identify transcriptional activators responsible for the tissue-specific expression of genes defining liver development and function [9]. In doing so, they discovered a new DNA-binding protein, HNF-3α, whose expression, along with C/EBP-β, was enriched in liver tissue and essential for transcription of the liver-specific genes transthyretin (*Ttr*) and α1-antitrypsin (*Serpina1*), and liver morphogenesis [9]. This seminal study spurred the realization that no single transcription factor dictated cell identity, but rather networks of tissue-specific transcription factors controlled developmental programs of gene expression (reviewed [10]). Around the same time and on the other side of the globe, the *Drosophila* forkhead (*fkh*) DNA-binding protein was determined to be critical for fly development by Weigel et al., and the sequence for the *fkh* gene was determined [11]. Alignment of the *fkh* and *Hnf3a* sequences revealed a 100-amino acid region of high homology encompassing the DNA-binding domain of the two factors [12]. This region lacked homeodomain and zinc-finger motifs, distinguishing it from known transcriptional regulators, uncovering a new class of transcription factor with unique properties [13].

Structural analysis of the related forkhead transcription factor HNF-3γ complexed with DNA revealed a DNA-binding region consisting of a helix-turn-helix motif, comprising three α-helices and two β-strands flanked by two polypeptide loops (designated as Wing1 and Wing2 subdomains). It was noted that the entire DNA-binding region resembled “a butterfly perched on a straight rod,” thus instilling the moniker “winged helix” to this new transcription factor motif [14,15]. Further studies revealed that HNF-3γ binds DNA similarly to linker histones, with the “wings” making contact with the minor grooves of the DNA double helix similar to histones 1 and 5. The helix-turn-helix motif also contacts DNA, protruding into the major groove, stabilizing the chromatin interaction [16,17]. Unlike linker histones, which compact nucleosomes into higher-order structures, HNF3/forkhead proteins lack the basic amino acids required for DNA compaction, thus relaxing the chromatin and enabling additional transcriptional regulators to access DNA. This inspired the monikers “pioneering” or “licensing” factors to this newly discovered class of proteins [18]. Moreover, unlike linker histones, HNF3-α typically binds as a monomer to either an A(A/T)TRTT(G/T)RYTY consensus element or as a homodimer, recognizing a five basepair core *fkh* motif that is flanked by 2 FOXA1 half-sites, termed “DIV motifs” in a sequence-specific manner [19,20].

The discovery of this new class of DNA-binding proteins launched an era of extensive research focused on the transcriptional mechanisms underlying developmental tissue specification. This led to the discovery of dozens of factors with DNA-binding domains that were highly homologous to those of the mammalian HNF-3α and *Drosophila fkh* in wide-ranging species from invertebrates to humans as well as in diverse cell types [21,22,23,24]. As new factors were identified, they were given various names, though they were fundamentally similar proteins with homologous DNA-binding domains. A new classification system was proposed to systematize the nomenclature, and the FOX family of transcription factors was born [25]. To date, the mammalian FOX (abbreviated from Forkhead Box) family encompasses 19 subclasses ranging from FOXA-FOXS. FOXA1 (formerly HNF-3α) is the founding member of the FOXA subclass, along with FOXA2 and FOXA3 (formerly HNF-3β and HNF-3γ, respectively) [25].

## 3. FOXA1 and Mammary Gland Development

### FOXA1 and ER Are Coordinately Expressed during Mammary Development

Breast cancer can be considered a caricature of normal mammary gland development, and several critical developmental factors, such as ER, are also implicated in breast cancer progression [26,27]. Like ER, FOXA1 is expressed in discrete compartments during mammary gland morphogenesis. In the developing postnatal gland, FOXA1 expression is compartmentalized in the body cells of the terminal end bud that contains the luminal progenitor cells and is undetectable in the more primitive cap cells of the bud. As the gland develops, FOXA1 is expressed in the ductal epithelial cells of the virgin gland, mirroring the expression pattern of ER. While FOXA1 and ER are coordinately expressed in ductal epithelium, expression of both proteins is reduced in alveolar structures, further decreased during pregnancy, and undetectable with the onset of lobulo-alveologenesis. ER and FOXA1 expression is then gradually restored following involution [26,28,29].

To study the impact of FOXA1 on mammary gland morphogenesis, Bernardo et al. examined rudimentary ductal trees from embryonic mice with homozygous deletion of *FOXA1*. At birth, mammary glands in wild type and *FOXA1* null mice are morphologically similar [28]. However, FOXA1 null pups are growth retarded and die shortly after birth due to hypoglycemia and dehydration [30,31,32]. Thus Bernardo et al. used mammary gland transplantation approaches to assess the requirement of FOXA1 on postnatal mammary gland development. In this context, *FOXA1* null mammary anlagen failed to form outgrowths when orthotopically xenografted into cleared fat pads of wild-type syngeneic mice. Similar observations were obtained using renal capsule transplantation of *FOXA1* null embryonic fat pads containing rudimentary ductal trees. In this case the fat pads grew, but the ductal trees did not grow further into the fat pads, indicating that FOXA1 expression in the mammary epithelium is essential for postnatal mammary gland development. Notably, the outgrowth of FOXA1 null transplanted ductal trees remained stunted when assessed at late pregnancy. However, alveoli were observed decorating the ductal tree of *FOXA1* null outgrowths. Both wildtype and null glands contained lipid droplets and expressed milk proteins, suggesting that while FOXA1 is essential for ductal morphogenesis, it is dispensable for formation and differentiation of alveolar luminal epithelial cells. Moreover, glands with heterozygous deletion of *FOXA1* contained an increased number of alveoli compared to wildtype controls. Together, these data suggest that suppression of FOXA1 may be necessary for alveologenesis to occur. Additional studies using inducible models of FOXA1 ablation throughout the mammary gland (MMTV-Cre) and within the mammary epithelial cell population specifically (Krt14-Cre), confirmed the necessity of FOXA1 expression for ductal formation [33]. The lack of ductal development with loss of FOXA1 phenocopies *ESR1* null glands [26,27,29]. Bernardo et al. then went on to show that ER expression was undetectable with loss of FOXA1 [28]. These studies underscore the critical need of FOXA1 for ER expression and mammary gland development. 

## 4. FOXA1 and the ER-Controlled Transcriptome in Breast Cancer

### 4.1. FOXA1 and ER Expression Are Positively Correlated in Breast Cancer

ER expression is prognostic of favorable outcomes of breast cancer patients and, as mentioned above, is highly expressed in the luminal breast cancer subtypes [7,8]. Large-scale gene expression studies of breast cancer cell lines and primary tumor tissue were instrumental in identifying the selective upregulation of FOXA1 in luminal breast cancers and its strong correlation with *ESR1* expression [7,8,34,35,36]. Immunohistochemical analyses confirmed these findings, with 84% of ER-positive breast cancers having high FOXA1 expression [37]. The considerable correlation between FOXA1 and ER expression is not surprising given the requirement of FOXA1 for ER expression in normal mammary epithelium [28]. Notably, FOXA1 is also independently prognostic of improved outcomes of patients with ER-positive disease as well as response to endocrine therapy, supporting an essential function of FOXA1 in luminal breast cancer [7,8,38].

### 4.2. FOXA1 Is a Pioneering Factor for ER Binding to Chromatin

Robyr et al. were the first to demonstrate the synergistic association between the pioneering function of FOXA1 and ER transcriptional activation by evaluating nucleosomal DNA conformation in *Xenopus* oocytes. Their studies revealed that FOXA1 could bind to and relax compacted DNA encompassing the promoter of the well-characterized ER responsive gene, *Vitellogenin*. This binding facilitates ER access and rapid activation of transcription [39]. A similar induction of transcription was observed upon FOXA1 binding to the regulatory region of trefoil-factor 1 (*TFF1*), another established estrogen-responsive gene in mammalian cells [40]. With the advent of DNA microarrays and next generation sequencing, the impact of FOXA1 on ER transcriptional programs was rapidly expanded from individual genes to the whole genome. Using chromatin immunoprecipitation (ChIP) combined with tiled DNA microarrays targeting chromosomes 20 and 21, Carroll et al. discovered the broad-ranging requirement for FOXA1 to facilitate ER-binding and estrogen-induced transcriptional programs in a luminal breast cancer cell line [41]. The technical approach used in these studies revealed enrichment of ER binding events at distal enhancers compared to promoter-proximal regions of ER target genes. Moreover, roughly half of the ER-bound sites overlapped with FOXA1-binding sites [41,42]. Interestingly, these studies and others found that FOXA1 binding occurs at most ER-binding regions before ligand treatment [38,41,43,44,45,46,47]. Thus, antecdent FOXA1 binding is required for ligand-induced ER chromatin association and transcriptional activation of ER-regulated genes, a plausible finding given prior studies demonstrating that FOXA1 relaxes compact chromatin. Together, this work revealed that FOXA1 is essential for activation of chromosome-specific, ER-dependent transcriptional programs in luminal breast cancer. Subsequent genome-wide analyses have confirmed these findings, with some exceptions in the number of overlapping ER-FOXA1 motifs and enrichment at proximal promoter regions [42,48]. Importantly, FOXA1 binding is not blocked with ER-inhibition, suggesting the FOXA1 cistrome is independent of ER activation by its ligands [38,46].

It is now well established that the overwhelming majority (>90%) of estrogen-regulated genes require FOXA1 for estrogen response, as FOXA1 silencing results in global inhibition of ER association with chromatin and transcriptional activity [38,42,44,46,49]. In contrast, only ~50% of ER-binding events precisely overlap with a FOXA1 binding event. Two possible scenarios can explain these differing observations. First, a distant FOXA1-binding event stabilizes the binding of ER to a site that is more proximal to the gene. Second, FOXA1 stabilizes or induces a secondary factor that subsequently promotes ER binding to its target sites. Of note, GATA3, another pioneering factor upregulated in ER-positive breast cancers, collaborates upstream of FOXA1 to promote ER-transcriptional programs and is also required for ER response [50]. However, FOXA1 is sufficient for ER chromatin occupancy and activation of breast cancer signature genes in non-breast cancer cells that typically have low/undetectable FOXA1 expression (i.e., osteosarcoma cells) [38]. As FOXA1 governs the estrogen-regulated transcriptome and cell growth in breast cancer, it is unsurprising that FOXA1 is required for estrogen-induced cyclin D1 (*CCND1*) expression, cell cycle progression, and proliferation of luminal breast cancer [45]. Together, these studies indicate that FOXA1 binding is essential for genome-wide chromatin remodeling, enabling ER access and engagement with cis-regulatory elements. Hence, FOXA1 is a pioneering factor for ER-stimulated gene expression in breast cancer cells [38,42,45,46].

### 4.3. FOXA1 Recruits Corepressors to ER-Repressed Genes

While estrogen-bound ER can directly induce gene expression, it can also repress transcription of target genes [41,51]. Since FOXA1 is required for ER-induced transcription, it is logical to surmise that it may also control ER-repressive functions. Whole-genome analyses for FOXA1 and ER binding using ChIP-chip and ChIP-Seq in the presence or absence of estrogen stimulation confirmed that both estrogen-induced and estrogen-repressed genes are significantly enriched for overlapping FOXA1 and ER binding events [46]. As with estrogen-induced genes, FOXA1 binds to enhancer regions of estrogen-downregulated genes prior to ligand exposure [46]. Several corepressors have been shown to mediate the repressive effects of FOXA1 at estrogen-repressed genes. HDAC7 (histone deacetylase 7) interacts with FOXA1 and ER, forming a ternary complex required for estrogen-directed repression of the cell cycle inhibitor, *RPRM* [52]. Another corepressor, TLE3 (TLE-family member 3), interacts with chromatin indirectly by binding to FOXA1 in the absence of estrogens at ER-responsive genes. TLE3 then recruits HDAC2 to repress gene expression [53]. Roughly half of the ER-FOXA1 binding events in the luminal MCF7 cell line are associated with TLE3, suggesting that FOXA1 and TLE3 together prevent expression in the absence of ligand [53]. Indeed, FOXA1 recruits TLE3 to chromatin to repress transcription of the estrogen-responsive gene, TFF1 [53]. Thus, FOXA1 acts as a bona fide pioneering factor to establish ER binding patterns in breast cancer, making FOXA1 a key determinate of hormonal response in this disease [38,41,43,44,46,47,49,54,55].

### 4.4. NRs May Reciprocally Act as Licensing Fators for FOXA1

While the pioneering function of FOXA1 in estrogen-driven breast cancers has been extensively reported, two studies directly challenge this paradigm. Caizzi et al. found that transient ER silencing in unstimulated MCF7 cells resulted in a significant decrease in FOXA1 binding events in close proximity to ER-bound sites, suggesting that ER binding can recruit FOXA1 to chromatin [56]. Furthermore, Swinstead et al. demonstrated that both ligand-bound ER and GR could reprogram the chromatin landscape in breast cancer cells and then recruit FOXA1 to a subset of binding sites that were inaccessible to FOXA1 prior to ligand stimulation [57]. These studies indicate that ER and GR can act as initiating factors for FOXA1 binding to chromatin, suggesting that activated NRs can reciprocally reprogram FOXA1-chromatin association. Swinstead et al. further found that FOXA1 has short dwell times when bound to chromatin, providing flexibility in its rapid binding response upon ligand stimulation. This contrasts with previous reports demonstrating that FOXA1 penetrates and forms stable interactions with condensed nucleosomes, relaxing chromatin and increasing accessibility to NRs [57,58,59]. DNAse hypersensitivity mapping for FOXA1 binding failed to yield a detectible footprint, suggesting FOXA1 chromatin occupancy is transient and highly dynamic [57]. This notion was supported by single-molecule tracking studies, demonstrating that FOXA1 residence times on DNA were short-lived, indicating that FOXA1 binding is more malleable than previously appreciated [57]. Glont et al. repeated key ChIP-Seq studies reported by Swinstead et al. to determine the impact of activated ER on the FOXA1-cistrome, using two different FOXA1 antibodies in two luminal cell lines and in the presence or absence of estrogen treatment [49]. They found that FOXA1 binding events induced by ligand-bound ER only constituted a minority (0.02 and 1%) of all FOXA1-binding, confirming previous reports that FOXA1 chromatin association precedes ER-binding affirming that FOXA1 chromatin occupancy is mainly unaffected by hormone treatment [49]. As a result, Glont et al. proposed that the hormone-induced reprogramming reported by Swinstead and colleagues was likely due to at least two factors: (1) high variability between ChIP-Seq replicates and (2) artifactual binding peaks resulting from ChIP fixation methods, which cross-link chromatin loops and create shadow FOXA1 binding sites that are far from consensus motifs [49,57]. To resolve this controversy, mutation of FOXA1 and NR consensus binding motifs, coupled with hormone treatment and ChIP-Seq analyses, may illuminate the timing of binding events within a biological context. Regardless of whether NRs can also serve as recruiting factors for FOXA1, the weight of evidence supports FOXA1’s role as a pioneer factor for estrogen signaling in breast cancer.

## 5. FOXA1 Dictates Cell Identity through ER Dependent and Independent Transcriptional Programs

### 5.1. FOXA1 Grooms the Epigenetic Landscape to Facilitate ER Binding

The ER-regulated transcriptome is the primary driver of luminal cell identity in both breast development and cancer. Hence the ability of FOXA1 to directly control the chromatin occupancy of ER also ultimately confers the luminal cell fate. In addition, FOXA1 is required for ER expression and normal mammary morphogenesis in mice [28], providing an additional mechanism by which FOXA1 ensures control of the ER-regulated transcriptome in the breast. Notably, FOXA1 also facilitates AR-chromatin association in prostate development and cancer as well as in breast cancer cells [46,60,61]. Since FOXA1 binding precedes ER and AR chromatin engagement and transcriptional programs, these data suggest that FOXA1-regulated cistromes drive the epithelial cell identity of these two organs through tissue-specific NR recruitment. Importantly, FOXA1 consensus motifs do not differ between breast and prostate epithelia [46]. Instead, the chromatin landscape provides a roadmap for cell-specific FOXA1 binding in the form of epigenetic modifications. Epigenetic changes, such as DNA methylation and post-translational histone modifications contribute to chromatin remodeling and facilitate the accessibility of transcriptional cofactors at regulatory regions, thus influencing transcriptional activity [62]. Distal enhancers that are poised for transcriptional activation are defined by an enrichment of H3K4 mono (me1) and dimethylation (me2). Notably, FOXA1-NR binding is enriched at distal enhancer regions in both breast and prostate in a cell type-specific manner [46,63,64,65]. These epigenetic marks are required for FOXA1 engagement with DNA tissue-specific sites, as removing these marks by overexpressing the H3K4me1/2 demethylase, KDM1 decreases FOXA1 occupancy at many of these regions [46]. Other histone modifications can also elicit tissue-specific events. For example, FOXA1 binding to the estrogen-target gene *TFF1* is dependent on the insertion of the variant histone, H2A.Z, into promoter-associated chromatin by P400, supporting a role for epigenetic regulation in controlling cell identity [66]. Interestingly, ectopic FOXA1 expression in TNBC cells results in the acquisition of H3K4me1/2 marks at FOXA1 bound sites, indicating that FOXA1 may promote histone methylation rather than be recruited by it [65]. Supporting this possibility, subsequent studies revealed that FOXA1 is recruited to enhancer regions devoid of methylation in a cell-type-specific manner [65]. Simultaneously, FOXA1 induces methylation of H3K4 at enhancers. This stabilizes FOXA1 binding and stimulates the recruitment of additional transcriptional modulators. Moreover, FOXA1 can recruit the histone-lysine N-methyltransferase, MLL3, to facilitate the deposition of H3K4me1 histone marks, and as a result, demarcating active enhancer elements in luminal breast cancer cell lines [54]. Together, these studies revealed that FOXA1 expression drives an epigenetic switch that alters the chromatin landscape and drives cell identity [65].

### 5.2. FOXA1 Represses TNBC/Basal Gene Expression to Maintain Luminal Cell Identity

**Figure 1:**
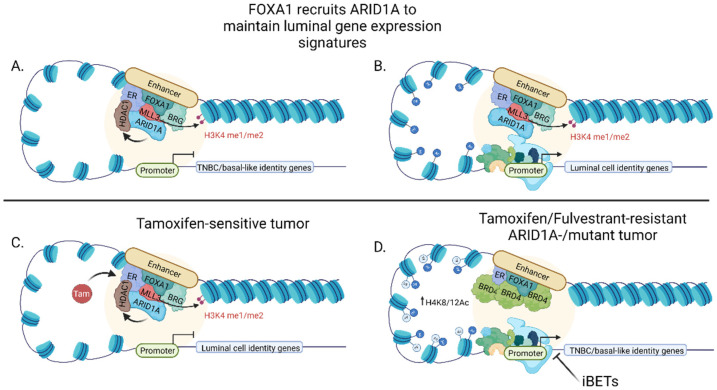
FOXA1 dictates luminal cell identity and response to endocrine-directed therapies in breast cancer. (**A**) FOXA1 represses expression of TNBC/basal-like signature genes and (**B**) facilitates luminal gene expression through recruitment of ER, transcription factors, and the SWI/SNF complex member, ARID1A; (**C**) FOXA1 and ARID1A are critical for endocrine-directed therapeutic response in breast cancer; (**D**) Therapy resistance driven by ARID1A mutation or loss is associated with acquisition of BRD4 binding and sensitivity to BET inhibition in breast cancer. Created with BioRender to represent findings published by [45,58,59,60,61,62].

Just as FOXA1 instructs luminal cell identity by defining the ER-transcriptome, FOXA1 also represses the expression of a subset of genes in luminal cell lines that are typically expressed in a more primitive breast epithelial lineage and are associated with more aggressive, triple negative breast cancers (i.e., TNBC/basal-like). This occurs even in cell lines that lack ER expression [67]. FOXA1 also prevents aggressive features normally observed in TNBC/basal-like cells, as transient FOXA1 silencing in luminal cell lines results in increased migratory and invasive potential in vitro [67]. As mentioned above, FOXA1 does not possess intrinsic repressor activity. In this regard, FOXA1 recruits members of the ATPase SWI/SNF chromatin remodeling complex to DNA to repress TNBC/basal-like gene expression [68]. Of the three SWI/SNF complexes that exist (i.e., BAF, P-BAF, and ncBAF), members of the BAF complex have been most closely associated with ER transcriptional activity and chromatin remodeling. Specifically, the BAF subunits, BRG and ARID1A, directly bind ER in both cell line and patient tumors and are required to modulate ER transcriptional activity [69,70]. Notably, the ARID1A-containing BAF complex is recruited to chromatin by FOXA1, where it represses transcription of TNBC/basal signature genes independent of ER activation in both cell lines and patient samples (Figure 1A) [71]. Interestingly, ARID1A silencing also decreased luminal gene expression signatures, including reduced expression of FOXA1, GATA3, TFF3, and ER, among others. This suggests that ARID1A promotes the expression of luminal identity genes (Figure 1B) [68]. This is in agreement with observations by Bernardo et al., wherein FOXA1 silencing induced TNBC/basal gene expression, as well as decreased gene expression of a small subset of luminal genes, including TFF3 even in the absence of ER [67]. Furthermore, ARID1A loss results in decreased HDAC1 binding and increased TNBC/basal gene expression, suggesting a potential mechanism by which TNBC/basal-like genes may be repressed [71]. Combined, these data affirm that breast cancers reflect different stages of luminal epithelial development with the basal epithelial phenotype preceding the luminal cell fate. Moreover, they indicate that the luminal cell differentiation cascade requires the transcriptional and epigenetic repression of basal genes that are also signatures for TNBC.

Genes essential for cell identity and oncogenic transformation are regulated by clustered enhancer regions, termed “super-enhancers” [72]. Super-enhancers are defined by at least three features: (1) Unusually high levels of BET-protein and mediator binding (e.g., BRD4 and MED1), (2) an exceptional enrichment of H3K4-acetylation, and (3) lineage-specific transcription factor binding [73]. Super-enhancers encompass large protein clusters that bind to discreet chromatin regions. These clustered proteins further recruit cofactors and chromatin modifiers through protein-protein interactions and cooperative binding kinetics [72]. The accumulation of multiple cofactors promotes a physical phase-separation similar to a membrane-less organelle, compartmentalizing transcriptional activity at essential cell identity genes as well as oncogenes [74,75]. As FOXA1 and ER define luminal cell identity, it has been suggested that ER and a subset of its key luminal defining targets are associated with super-enhancers. However, unlike previously characterized SEs, which exist at lineage-specific genes at steady-state in other tissues (e.g., MYC in multiple myeloma [75]), luminal cell identity is actually defined by the inducible nature of ER by estrogen. Using MCF-7 cells as a luminal breast cancer model, Bojcsuk et al. revealed that ligand binding to ER induces its interaction with and activation of enhancers. Together with neighboring cofactor-bound sites, the ER-bound enhancers generated functional super-enhancers [76]. Importantly, FOXA1 was co-bound at super-enhancers that interacted with ligand-activated ER, independent of FOXA1 motif enrichment. Hence, FOXA1 may associate with super-enhancers through protein-protein interactions rather than binding to DNA [76]. A separate study confirmed these data, identifying super-enhancers at luminal identity genes, including *FOXA1*, *ESR1*, *GATA3*, and *SPDEF* in somatic cell fusions of basal and luminal cell lines displaying a dominant luminal phenotype [77]. Thus, FOXA1 is a critical component of activated ER super-enhancers that drive cell identity.

## 6. FOXA1 Mutations and Resistance to Endocrine Therapies

### 6.1. Introduction to Mechanisms of Endocrine Therapy-Resistance

Several classes of therapeutics are highly effective in blocking estrogen response in breast cancer. These therapies have led to durable cures in roughly 70% of patients, yet ~30% of women develop tumors that usurp endocrine-directed treatments, resulting in recurrent disease [78,79]. Several mechanisms of endocrine resistance have been characterized, including decreased ER expression in 10–20% of tumors and subsequent compensatory activation of other NRs as described below (see FOXA1 and AR) [80,81]. However, the majority of resistant tumors maintain ER expression in the presence of endocrine inhibition and continue to proliferate by either activating alternative pathways such as growth factor signaling, inducing drug metabolism and efflux, changing ER cofactor expression required for ER signaling (e.g., FOXA1), or by modulating ER-FOXA1 enhancer binding.

**Figure 2:**
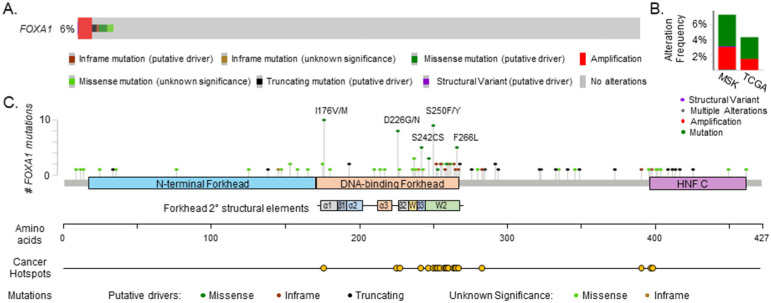
Amplification, mutation, and structural variation of the FOXA1 gene in breast cancer patients. (**A**) Oncoprint depicting *FOXA1* genetic alterations in 3002 primary and metastatic breast cancer samples (all subtypes) from 2840 patients analyzed by Memorial Sloan Kettering [79,82], and The Cancer Genome Atlas (TCGA PanCancer) [78], (**B**) *FOXA1* alteration frequency segregated by study, (**C**) Lollipop plot showing *FOXA1* mutations and clustered hotspot mutations in the *fkh* DNA-binding domain of *FOXA1* in MSK and TCGA samples and adapted from [79]. Data analysis was conducted with www.cbioportal.org accessed on 12 August 2021.

Tamoxifen is the oldest ER inhibitor still widely used, although it is progressively being replaced with aromatase inhibitors as first-line therapy in postmenopausal women with luminal breast cancer. Classified as a selective estrogen receptor modulator (SERM), tamoxifen does not completely block ER function. Rather, tamoxifen-bound ER is recruited to chromatin and represses transcription of ER target genes [82]. In tamoxifen-sensitive breast cancer cells, tamoxifen-ER binding events significantly overlap (93%) with those of estrogen-bound ER, and likewise, tamoxifen-bound ER requires FOXA1 for chromatin engagement and inhibitory action [38,48]. In contrast, ER binding patterns differ substantially in tamoxifen-resistant breast cancers, and the tamoxifen-resistant ER cistrome is prognostic of patient survival [38,83]. Interestingly, focal amplification and overexpression of FOXA1 is observed in several endocrine-resistant cell line models. Demonstrating an impact of increased FOXA1 levels, enforced FOXA1 overexpression substantially increases the number of chromatin-wide sites that bind to FOXA1 and enhances the strength of ER binding [38,84]. These data suggest that amplification or overexpression of *FOXA1* may drive endocrine-resistant disease. Several large-scale genomic analyses of patient tumors reinforce this possibility, both for tamoxifen and aromatase-inhibitor resistance. Focal amplification and hotspot mutations in *FOXA1* occur in 3–6% of breast tumors and are associated with endocrine resistance (Figure 2A,B) [85,86,87,88,89,90,91]. Deep sequencing of 300 primary breast cancers revealed recurrent hotspot mutations in the *FOXA1* promoter that coincide with increased expression [92], suggesting that these mutations drive elevated transcription of *FOXA1*.

### 6.2. Elevated FOXA1 Expression Reprograms the ER Cistrome in Endocrine-Resistant Disease

Mechanistically, it has been proposed that elevated FOXA1 levels rewire ER-dependent transcriptional programs and activate oncogenic signaling pathways to bypass ER blockade, promoting endocrine therapy resistance and disease aggressiveness [84,93]. FOXA1 overexpression in luminal breast cancer cell lines fundamentally alters the enhancer landscape, activating transcriptional programs associated with oncogenic signaling pathways similar to those observed in clinical breast cancer specimens [93]. Moreover, enhancer regions selectively activated in metastatic breast cancer are associated with FOXA1 transcriptional programs [94]. Using five tamoxifen-resistant cell line models, Fu et al. confirmed amplification or overexpression of FOXA1 is associated with genome-wide FOXA1-ER transcriptional reprogramming. This differential chromatin occupancy induced proliferative and invasive properties consistent with metastatic tumor cells [84,93]. Thus, hyperactive FOXA1 drives endocrine-resistant breast cancer phenotypes through transcriptional reprogramming. Such reprogramming extends to the acquisition of super-enhancers during tumorigenesis [95]. Super-enhancer activation is also associated with endocrine resistance in luminal breast cancer. Moreover, tamoxifen-resistant cell lines harboring amplification of the *FOXA1* gene acquire super-enhancers that are not identifiable in the parental cell lines [93]. The most notable super-enhancer acquisition is at the *HIF2A* gene, which, along with increased FOXA1 expression, stimulates the expression of downstream genes associated with highly motile metastatic phenotypes, including those involved in extracellular matrix organization, focal adhesion, and angiogenesis pathways [93]. Furthermore, inhibition of HIF2A with selective antagonists decreased metastatic phenotypes observed in endocrine-resistant cells, providing early preclinical evidence for using HIF2A inhibitors in resistant disease [93].

### 6.3. FOXA1 Mutations Confer Endocrine Therapy Resitance

In addition to focal amplifications, the *FOXA1* locus is subject to hotspot mutations (Figure 2C) [88,91]. A recent examination of *FOXA1* mutations in >4950 breast tumors and metastatic lesions found that 4.18% of breast cancers and 4.88% of metastases harbored recurring mutations, with the majority occurring in the Wing2 subdomain, consistent with previous reports [88,89,90]. *FOXA1* missense mutations are enriched in metastatic luminal breast cancers compared to primary lesions and are often mutually exclusive with mutations in *ESR1* [96,97,98]. These mutations are clinically significant as patients with *FOXA1* mutant tumors receiving aromatase inhibitors have shorter progression-free survival than those with wild-type FOXA1 [88]. Clustered activating mutations in *FOXA1* have been classified into three categories in both breast and prostate cancers [85]. Class I missense and indel mutations are the most frequent *FOXA1* mutations observed in breast cancer [88]. These missense mutations are localized in the Wing2 region of the *fkh* domain. They enhance FOXA1 binding to DNA, induce chromatin remodeling and stimulate ER-dependent transcriptional programs [85]. Of note, class I *FOXA1* mutations are also more frequently associated with invasive lobular breast cancer than ductal disease and may suggest a different role for these mutations in different breast cancer subtypes [86]. Class II activating mutations cluster in the c-terminal C2 domain and result in truncation of FOXA1. These mutations also enhance FOXA1 association with DNA but reduce its interaction with TLE3, a transcriptional corepressor of Wnt signaling in prostate cancer cells [85]. Lastly, class III mutations consist of structural rearrangements and duplications within the *FOXA1* locus, resulting in aberrant FOXA1 regulation and increased expression. These mutations are clinically important as patients with *FOXA1* mutant tumors receiving aromatase inhibitors have shorter progression-free survival than those with wild-type FOXA1 [88]. Mechanistically, mutations in the Wing2 domain confer the highest gain in proliferative capacity compared to those in other domains when assessed in the absence of estrogen in vitro and xenografts into mice. Wing2 mutant FOXA1 also exhibits stronger genomic binding at canonical ER target genes with estrogen stimulation. Unlike wild-type FOXA1, whose pioneering function is not influenced by estrogen stimulation, Wing2 FOXA1 mutants more strongly associate with DNA upon estrogen treatment. FOXA1 Wing2 mutations are also mutually exclusive with mutations in *ESR1*, yet the cistrome and transcriptome of Wing2 FOXA1 mutants mirror that of activating mutations of the estrogen receptor. These findings suggest that Wing2 mutants sustain the active ER program to stimulate endocrine-resistant disease. 

In contrast to Wing2 mutations, which drive a hypermorphic estrogen response, an SY242CS mutation in the beta-strand of the helix-turn-helix domain actually decreased ER transcriptional activity when ectopically expressed in vitro [88]. Furthermore, ectopic expression of the SY242CS FOXA1 variant altered the chromatin landscape, revealing an enrichment of a non-canonical FOXA1 DNA-binding motif (TTTA/GTTTA/G) and conferring a significantly altered transcriptome that is independent of estrogen treatment and suggestive of a novel pioneering function of this variant. Furthermore, unlike Wing2 mutants, the SY242CS-driven transcriptome shows a negative enrichment of estrogen-response signatures, suggesting that this mutant drives an alternative pathway to endocrine resistance. Of note, all *FOXA1* variants tested in these studies were sensitive to fulvestrant (SERD) and tamoxifen (SERM) treatment, suggesting that *FOXA1* mutations specifically contribute to aromatase inhibitor resistance. In contrast, mutations in *ESR1* mutations more broadly drive resistance to all endocrine therapies. The mechanisms underlying these differences are not yet clear but likely relate to the differing functions of FOXA1 (licensing) versus ER (transcriptional activation).

### 6.4. ARID1A Mutations Induce Endocrine Therapy Resistance by Shifting Tumor Cell Identity

Mutations in SWI/SNF chromatin remodeling complex can also drive endocrine resistance. Strikingly, breast cancer patients with tumors harboring *ARID1A* mutations have a significantly reduced progression-free survival when treated with ER-degrading therapies compared to wild-type *ARID1A* tumors [68]. Mechanistically, tamoxifen treatment of breast cancers with wild-type ARID1A results in recruitment of HDAC1 to ARID1A-FOXA1-ER bound enhancers, resulting in decreased ER activity (Figure 1C). In contrast, tumors with mutant ARID1A lose HDAC1 binding and gain accumulation of BRD4 and increased H3K4/8 acetylation at ER-bound enhancers, conferring increased proliferation (Figure 1D) [71]. Notably, this renders ARID1A-mutant tumors susceptible to BET inhibition, providing a potential avenue for usurping endocrine-therapy resistance in this context (Figure 1D) [71]. Furthermore, ARID1A mutant tumors express gene signatures associated with basal-like disease, consistent with the impact of *ARID1A* loss in cell lines [68,71]. Combined, these studies reveal that the pioneering function of FOXA1 is a precipitating event, recruiting activated ER to cis-regulator regions, as well as cofactors that facilitate epigenetic modifications. Together, these factors define luminal cell identity. FOXA1 also serves as a rheostat for estrogen signaling, maintaining basal gene expression in the absence of ligand. Moreover, disruption of FOXA1 expression or function can shift the balance of luminal cell identify to either a more primitive, TNBC/basal-like phenotype or a luminal cell that is independent of estrogen signaling, both of which convey aggressive disease features.

## 7. Progesterone Cistromes Are Largely Independent of FOXA1

### 7.1. PR Chromatin Occupancy Is Independent of FOXA1

Expression of ER and PR are the two most defining features of luminal breast cancer. While not therapeutically targeted in this disease, PR is a direct transcriptional target of ER. As such, its expression is used as a surrogate marker of ER activity and breast cancer prognosis. Breast cancers with strong PR expression are associated with improved patient outcomes, as they reflect a tumor driven by active ER signaling and intrinsic sensitivity to endocrine therapies [99,100,101]. Progesterone activates transcription through two distinct mechanisms. A small fraction of PR is bound to the inner cytoplasmic membrane where, upon ligand binding, it rapidly associates with, and is phosphorylated by, MAPK [102]. Together with MSK1, PR and ERK bind chromatin to activate gene transcription [103,104]. In contrast to this rapid signaling effect, the majority of PR binds DNA directly at PR response elements. PR then recruits histone-modifying enzymes and ATP-dependent chromatin remodelers, such as NURF and SWI/SNF complexes (i.e., BAF complex) to engage additional cofactor binding [105]. Like ER, most PR binding regions are located in distal enhancers demarcated by histone modifications [106]. While the pioneering function of FOXA1 is essential for ER transcriptional programs, there is limited evidence that it is also required for PR cistromes. A comparison of PR cistromes between normal breast and breast cancer cell lines uncovered an enrichment of FOXA1 consensus elements near PR binding sites in breast cancer compared to normal breast [106]. However, Ceballos-Chávez et al. demonstrated that progestin-bound PR associates with DNA independently of FOXA1 and then recruits CHD8 (chromodomain helicase DNA binding protein 8) [107]. Like FOXA1, CHD8 can remodel nucleosomes to facilitate transcription factor binding; however, it does so in an ATP-dependent fashion [108,109]. Depletion of select SWI/SNF complex ATPases (i.e., BRG1 and BRM) reduced CHD8 recruitment, suggesting that ATP-dependent mechanisms may facilitate PR chromatin occupancy through CHD8 [107]. These data indicate that PR activation of transcription involves a very different mechanism than ER.

### 7.2. PR Activation Directly Shifts the ER-Cistrome Independent of FOXA1

Several studies have also demonstrated an antiproliferative effect of progesterone in luminal breast cancer cells lines and patient-derived xenografts (PDXs) that have strong PR positivity, indicating that PR is more than a biomarker of breast cancer outcome. PR is recruited to ER-bound chromatin upon exposure to combined estrogen and progesterone [110]. Dual receptor activation shifts ER-chromatin occupancy sites, inducing a shift from an estrogen-induced proliferative program to a more differentiated and less proliferative phenotype. Motif analysis of the PR-induced ER cistrome revealed enrichment of FOXA1 consensus sites, suggesting that FOXA1 expression levels dictate PR-driven cell identity shifts associated with ER [110]. However, Singhal et al. found that PR-induced reprogramming of ER chromatin occupancy and transcriptional programs did not require FOXA1 for nucleosomal remodeling, even though FOXA1 motifs were in close proximity [111]. In the presence of both estrogen and progesterone, PR was directly bound to ER. Indeed, only these two proteins were found to interact in pull-down assays under these conditions, suggesting PR may be a critical cofactor of ER in conjunction with ATP-dependent chromatin remodeling enzymes [107,111]. Combined, these studies nominate PR as a direct transcriptional modulator of ER function and breast cancer outcomes, likely in a FOXA1-independent manner.

## 8. FOXA1 Is a Pioneering Factor for AR in Luminal Breast Cancer

### 8.1. Ligand-Activated AR Reprograms FOXA1 Binding in Luminal Breast Cancer

Like ER, androgen receptor (AR) is expressed in the majority (~85%) of breast cancers and is positively correlated with FOXA1 expression, yet its role in breast cancer is less understood [112,113,114,115,116,117,118]. AR is a well-established oncogenic driver and therapeutic target in prostate cancer, and the requirement of FOXA1′s pioneering function for AR activation and disease progression is well established (reviewed in [119]). In contrast, the role of AR in breast cancer is controversial, with studies advocating for the use of both AR agonists and antagonists for treating naïve and resistant disease [120,121,122,123]. Adding to the complexity, AR expression is prognostic of both improved and worse patient outcomes depending on protein vs. mRNA detection platforms, discrepancies in binning of expression levels, breast cancer subtype, and clinical endpoints (reviewed in [122,124]). Most studies in luminal breast cancers found that AR expression is associated with less aggressive tumor characteristics and extended patient survival [81,125,126,127]. Interestingly, treatment with AR agonists in this subtype disrupts ER-signaling and proliferation, supplanting ER at the chromatin and driving AR signaling [81,117,121,127,128,129,130]. Indeed, a recent study found that ligand activation of AR profoundly decreases tumor burden in cell lines and mouse models of patient-derived luminal breast cancer, including those of endocrine and palbociclib (CDK4/6 inhibitor) resistant disease [121]. Ponnusamy et al. demonstrated similar findings using AR agonists or enobosarm, a selective androgen receptor modulator (SARM), in PDXs and tissue explants expressing either wild-type or mutant ER [131]. AR antagonism had no effect, reinforcing the importance of activating AR to suppress tumor growth. ChIP-Seq analysis of treated tumors revealed that activated AR inhibits growth by reprogramming ER chromatin occupancy. Interestingly, ER binding was decreased at ER-target genes in response to enobosarm, and a subset of ER binding events was enriched at androgen response element motifs. Similarly, enobosarm reprograms the FOXA1-cistrome, resulting in depletion of FOXA1 binding at ER-target genes and enrichment at enhancer regions bound by AR [131]. Overall, these studies demonstrate androgen-induced tumor regression in PDX models and thus provide a preclinical foundation for stimulating AR function to treat ER-positive breast cancer.

### 8.2. FOXA1 Redirects AR Binding to ER Consensus Sites in Molecular Apocrine Breast Cancer

In contrast to luminal disease, the role of AR in the context of ER-negative breast cancer is pro-tumorigenic [122,123,132,133]. In this context, AR activation stimulates proliferation and progression of HER2-enriched and TNBC subtypes, and combinatorial AR and HER2/mTOR inhibition synergistically reduces proliferation and tumor growth in mice [134]. Likewise, antagonizing AR function in combination with CDK4/6 inhibition (abemaciclib) suppresses TNBC tumor growth [135], the direct opposite of that observed for luminal disease where AR agonism synergized with CDK4/6 inhibition (palbociclib) to block growth [121]. Of note, while FOXA1 is selectively upregulated in luminal breast cancers, a significant proportion of TNBCs (~30%) also express FOXA1 [37]. AR is expressed in TNBC as well, albeit to a lesser extent. Moreover, FOXA1 expression is more highly correlated with AR than ER expression in breast cancer, suggesting the dual functions of FOXA1 in AR-positive versus ER-positive disease. A subclass of TNBC known as “molecular apocrine” cancers are defined by coexpression of AR and FOXA1 as well as a luminal breast cancer signature, despite lacking ER expression [136]. This group of tumors is also referred to as the luminal androgen receptor (LAR) subtype, in the TNBC subtype classification scheme [137,138,139]. MDA-MB-453 is a molecular apocrine cell line, and it requires AR signaling for proliferation and survival independent of estrogen receptor [136,140]. Given the well-established role of FOXA1 in mediating AR transcriptional programs in prostate cancer, it is logical to predict that these two proteins also cooperate in molecular apocrine breast cancer to promote AR activation and target gene expression. There are several lines of evidence supporting this hypothesis. First, mapping of AR binding sites in MDA-MB-453 cells revealed a landscape reminiscent of ER-binding [61]. In fact, the AR-binding profile in this cell line is more similar to the breast cancer ER-cistrome than AR-binding events in prostate cancer, indicating that FOXA1 directs AR binding to ER consensus sites. This property conveys a luminal gene expression signature despite the absence of ER [46,61]. However, in contrast to the ~50–60% overlap of ER and FOXA1 binding sites in luminal breast cancer cells, nearly all AR-binding sites are co-occupied by FOXA1 [61]. Furthermore, FOXA1 binding is required for AR to associate with chromatin and induce expression of the majority (~91%) of apocrine signature genes. On a functional level, cell proliferation and colony formation were significantly decreased with loss of FOXA1 [61]. These data indicate that the pioneering function of FOXA1 is essential for AR-driven cancers in the absence of ER. Of clinical relevance, patients with AR+/FOXA1+ TNBCs have worse overall survival than patients with AR−/FOXA1+ or AR−/FOXA1− disease [141]. Most importantly, single-agent AR-inhibition in patients with advanced AR-positive TNBC leads to superior patient outcomes. Thus, in TNBCs, AR plays an oncogenic role similar to that observed in prostate cancer [142]. Thus, while AR agonists may be effective in luminal AR+ breast cancer, these data support the use of AR inhibitors for the treatment of TNBCs that express AR.

### 8.3. The Complexity of Therapeutically Targeting AR in Endocrine Resistant Disease

As mentioned above, the majority of ER-positive breast cancers express AR. Thus, it is reasonable to expect that the development of resistance to estrogen/ER-targeting therapies may involve switching from reliance on ER to AR dependency for driving proliferation and progression. Thus, AR inhibitors may be effective in endocrine-resistant luminal breast cancer. Notably, silencing of AR expression, but not pharmacological inhibition, decreases proliferation of endocrine-resistant apocrine tumors, suggesting that non-canonical AR signaling may drive estrogen independence [120]. It is likely that the crosstalk between ER and AR is dependent upon ligand accessibility, expression of cofactors such as FOXA1, and the ratios of each NR. Given the complexities of AR modulation in breast cancer subtypes and the unclear functional relationship of AR and ER in those subtypes, further studies are necessary to fully understand how pharmacological manipulation of AR function may be most effective in the clinical setting.

## 9. FOXA1, Glucocorticoid Receptor, and Estrogen Receptor Dynamics

### 9.1. FOXA1 Is Essential for GR Chromatin Engagement in Luminal Breast Cancer

Like androgen receptor, the role(s) of the glucocorticoid receptor in breast cancer is not fully understood. Activation of the glucocorticoid receptor (GR) induces apoptosis in blood cancers, and synthetic glucocorticoids are widely used to treat hematologic malignancies. In contrast, breast cancer patients obtain little survival benefit from these agents, but are often prescribed glucocorticoids in conjunction with chemotherapy to prevent allergic reactions and alleviate symptoms of toxicity, such as nausea [143,144]. In addition to the desired systemic effects of glucocorticoids, it is important to recall that GR is expressed in 50–70% of breast cancers, is highly correlated with ER and FOXA1 expression [145], and is prognostic of favorable patient outcomes when examining all breast cancers as a group [146]. FOXA1 also plays a pioneering role for GR in breast cancer. This was first recognized in 2005 by Holmqvist et al., where FOXA1 was found to bind to heterochromatin encompassing the mouse mammary tumor virus (MMTV)-promoter and facilitating GR-mediated transcriptional activation [147]. In preclinical models of luminal breast cancer, GR activation inhibits estrogen-induced proliferation in the context of both ligands [148,149]. Ligand-activated GR displaces ER from chromatin at the regulatory regions of key pro-proliferative genes, including *CCND1*, *CDK2*, and *CDK6* [148,149]. FOXA1 is essential for GR binding to ER-bound regions [148]. As is the case for ER, GR-binding sites within chromatin are highly accessible prior to ligand activation [150]. Most GR binding events (69%) overlap with FOXA1-bound loci in luminal breast cancer cells devoid of glucocorticoids. Combined, these data indicate that FOXA1 binding precedes GR chromatin occupancy and likely acts as a pioneering factor for GR on its target genes [57]. However, treatment with the GR agonist dexamethasone enriches FOXA1 (and GATA3) binding at GR-binding sites, suggesting that FOXA1 binding can be reformatted with GR activation. This activity is dependent on the BRG subunit of the SWI/SNF complex [150]. A similar restructuring of FOXA1 binding was observed by Swinstead et al. [57], supporting the postulate that FOXA1 chromatin occupancy is dynamic, at least within the context of ligand-activated GR.

### 9.2. The Role of FOXA1 in Controlling the Function of GR in TNBC Progression and Therapy Resistance Has Not Yet Been Established

While glucocorticoids decrease the proliferation of breast cancer cells in vitro, growing evidence suggests that GR activation is associated with breast cancer progression in TNBC. GR activity is consistently increased in metastases of PDX models of this subtype of disease [151]. Moreover, dexamethasone promotes enrichment of EMT signature genes and metastatic colonization of the MDA-MB-231 TNBC cell line in mice [151]. Mechanistically, activation and phosphorylation of GR promotes induction of stress-associated genes (e.g., Brk and HIFs), leading to increased cell survival and resistance to chemotherapy [152,153]. In patients, high GR expression is associated with a shorter time to recurrence in both chemotherapy naïve and treated early-stage disease, suggesting that GR promotes breast cancer progression and chemotherapy resistance in TNBC patients [154]. Despite these data and the established role of FOXA1 in governing ER and AR transcriptional programs, little is known regarding the impact of FOXA1 on GR target genes in this breast cancer subtype.

## 10. FOXA1 Remodels Chromatin to Enable RAR Binding

Retinoic acid ligands (RA), such as all-trans retinoic acid, bind to receptors in the RAR nuclear receptor subclass, which includes RARα, RARβ, and RARγ. These receptors bind to DNA as heterodimers with the retinoic X receptor (RXR), where they repress transcription in the unliganded state. Upon ligand binding, RAR-RXR heterodimers can either induce or repress transcription of RAR target genes. RA is a potent inhibitor of breast cancer cell proliferation and inducer of apoptosis in pre-clinical studies. However, RA has failed as a therapy in breast cancer when used in combination with tamoxifen. This is likely due to crosstalk between RAR and other NR, such as ER and PR, and FOXA1 [155,156].

Just as FOXA1 acts as a licensing factor for ER chromatin binding, FOXA1 is also required for recruitment and binding of RAR to DNA in breast cancer cells. Using ChIP-chip studies assessing chromatin association of GFP-tagged RARα and RARγ expression constructs, Hua et al. found that silencing FOXA1 expression reduced RAR chromatin binding at shared RAR/FOXA1 binding regions, but the loss of FOXA1 had no impact on RAR binding at sites not shared with FOXA1 [156]. Furthermore, FOXA1 loss reduced the expression of RA-target genes that were co-bound by RAR and FOXA1, supporting the requirement of FOXA1 for a subset of RAR transcriptional targets. RAR/FOXA1 shared sites also overlapped with ER binding, suggesting crosstalk between RAR and ER signaling pathways. Interestingly, treatment with RA repressed the transcription of these genes while estrogen-treatment induced target gene expression, implying that these two pathways are antagonistic. Notably, FOXA1 is also a direct transcriptional target of RAR. RA-treatment induces FOXA1 expression and RAR directly binds to the FOXA1 gene [156]. Thus, the interplay of FOXA1 and RAR is complex, with RAR controlling the function and expression levels of FOXA1.

Endogenous RARα chromatin association has also been assessed in cell lines and patient samples by Ross-Innes and colleagues. Here, the authors demonstrated that RARα is a direct transcriptional target of ER and is required for ER-induced cell cycle progression and proliferation in the presence of estrogens [157]. ChIP-Seq studies revealed that like ER, RARα also preferentially binds to enhancer regions across the genome. Interestingly, roughly half of all RAR binding events overlap with those of ER, where enrichment of both RAR and ER motifs are found. Furthermore, inhibiting ER expression reduced RARα chromatin binding to these shared regions, demonstrating a requirement of ER for RAR binding, as well as ER-regulated gene transcription. In contrast, ER binding did not require RAR [157]. These two studies demonstrate that RARα can participate as a member of the FOXA1-ER-transcriptional complex and is required for transcription of a subset of ER-target genes. Notably, treatment with RA causes RAR to supplant ER binding at sites that are also bound to FOXA1. This promotes expression of RAR target genes and inhibits ER-mediated cellular proliferation. These data provide a rationale for using RAR-agonists as breast cancer therapies. However, early phase clinical trials with RA in combination with tamoxifen did not improve patient outcomes and it has been postulated that RAR crosstalk with other receptors, including PR, may actually enrich the number of tumor cells with more aggressive properties [155,156]. Further studies assessing NRs and FOXA1 crosstalk in the context of the complete and varied hormonal milieu are needed to reveal the relative contributions of NRs in physiologically relevant models of both ER-positive and ER-negative breast cancer.

## 11. NR and FOXA1 Chromatin Occupancy in Male Breast Cancer

As in the majority of women, male breast cancers are driven by ER transcriptional programs. Severson et al. leveraged the differing hormonal milieus present in male and female breast cancer patients to assess genome-wide binding of ER, AR, PR, and GR, along with FOXA1, GATA3, and the enhancer-enriched histone mark, H3K4me1, in breast tumors from both genders [158]. These studies revealed that all factors preferentially bind to intergenic and enhancer regions, consistent with previous reports in both male and female breast cancer. Notably, the vast majority of ER and FOXA1 binding sites were shared between male and female diseases. However, ER binding sites that are prognostic of survival in female breast cancer patients were not associated with outcomes of male breast cancer patients. However, differences in ER and FOXA1 cistromes within male breast cancers were capable of segregating patient outcomes, suggesting that FOXA1 may influence epigenetic regulation of chromatin in a gender-specific manner. In contrast to ER, PR binding sites, which affect ER chromatin occupancy in female breast cancers, were largely devoid of concordant ER binding in male cancers. These data suggest that, like FOXA1, PR may also have gender-specific functions in breast cancer.

## 12. Nuclear Receptor-Independent Functions of FOXA1

### 12.1. An ER-Independent FOXA1 Cistrome

There is growing evidence suggesting that FOXA1 has a role independent of ER and other nuclear receptors in breast cancer. As described above, FOXA1 represses TNBC/basal signature genes in cell lines lacking ER. While AR is expressed in the MDA-MB-453 cell type examined in these studies, the SKBR3 cell line lacks ER, PR, and AR expression. Indeed, up to 80% of FOXA1 binding events in luminal breast cancer cells do not overlap with ER-bound chromatin, independent of estrogen treatment. This is also true in estrogen-rich media, where most FOXA1 binding sites do not overlap with ER binding, suggesting ER-independent functions of this transcription factor [53].

### 12.2. FOXA1 Expressed in Tumor Cells Suppresses the Tumor Immune Response

Recently, a role for FOXA1 that is independent of its pioneering function was discovered in the regulation of the tumor immune response. Immuno-oncology is a rapidly expanding field and the application of immune checkpoint inhibitors for the management of aggressive cancers, such as melanoma and lung malignancies, has greatly extended patient outcomes by reactivating exhausted tumor-infiltrating lymphocytes (TILs) and re-engaging an anti-tumor immune response (reviewed in [159]). While checkpoint inhibitors are effective in other tumor types, most breast cancers, especially those with elevated expression of FOXA1, are immunologically “cold,” precluding any benefit from the use of immune checkpoint inhibitors. Interferon (INF) signaling pathways (including INFα, INFβ, and INFγ) are required for the anti-tumor immune response and subsequent cancer cell death, and tumors with low INF activity are often resistant to immune checkpoint inhibitor therapies [160,161]. A recent study revealed that FOXA1 expression is negatively correlated with interferon activity and the INF-response gene expression signature in luminal breast cancer, suggesting that FOXA1 suppresses the immune response in this disease [162]. Supporting this, neoadjuvant chemotherapy-treated tumors that express high levels of FOXA1 have low INF activity, reduced pathological response to chemotherapy, and decreased immune response [162]. Transcriptional induction of INF and its target genes is mainly dependent on STAT (signal transducer and activator of transcription) signaling that is independent of ER binding [163]. However, FOXA1 can directly bind to the DNA-binding domain of STAT2. This sterically impedes the interaction of STAT2 with interferon consensus elements in DNA and abrogates transcription of interferon target genes. As a result, increased FOXA1 suppresses the tumor immune response. Interestingly, FOXA1 motifs were not detected in close proximity to STAT binding, nor did FOXA1 bind to and remodel the DNA, indicating a function for FOXA1 that operates independently of its pioneering function for nuclear receptors.

### 12.3. FOXA1 Is Required for Lapatinib-Resistance in HER2-Enriched Breast Cancers

FOXA1 also has an NR-independent function in HER2-enriched breast cancers, where it is expressed in ~70% of this tumor type. HER2 signaling upregulates the expression of FOXA1 through ERK signaling, as well as activation of CREB1, AP2α, and cFOS, which bind and activate the transcription of the *FOXA1* gene [164,165]. FOXA1, in turn, stimulates transcription of HER2-regulated genes in a feed-forward loop, revealing crosstalk between receptor tyrosine kinases, such as HER2, with FOXA1 in breast cancers that lack ER expression. Indeed, silencing FOXA1 expression in HER2-enriched breast cancer cell lines inhibits proliferation, independent of ER expression [166]. Notably, increased expression of FOXA1 is also associated with therapy resistance in this breast cancer subtype. Mechanisms of acquired lapatinib (EGFR/HER2 inhibitor) resistance are diverse, including expression of truncated/activated HER2 and elevated PI3K/AKT signaling that lead to dramatic epigenetic and transcriptional changes. Angus et al. found that a large number of superenhancers are acquired in the context of lapatinib resistance, including those at the ERBB2 (HER2), PBX1, and FOXA1 genes [167]. They further revealed that the increased expression of FOXA1 is essential for proliferation of lapatinib-resistant cell lines and, in a feed-forward manner, induced the expression of ERBB3 (HER3). Demonstrating the importance of this loop, silencing FOXA1 expression in combination with HER2-inhibition decreased proliferation of lapatinib-resistant cells, independent of ER expression. These results underscore the requirement for FOXA1 in lapatinib-resistant, HER2+ disease. Patient data support this conclusion where breast tumors with decreased expression of *FOXA1* were less proliferative, had reduced *ERBB2/ERBB3* (HER2/HER3) expression, and increased enrichment of immune signatures [167]. As a whole, these studies revealed that the function of FOXA1 extends beyond its canonical role of modulating ER activity in breast cancer. Future studies will likely reveal additional mechanisms by which FOXA1 controls the expressed genome both in normal and tumorigenic breast epithelium.

## 13. FOXA1 as a Therapeutic Target in Breast Cancer

Broadly, FOXA1 facilitates proliferation and differentiation of luminal breast cancer cells through modulation of NR function. Conversely, FOXA1 suppresses more primitive tumor cell phenotypes, such as migration and invasion. Therefore, inhibiting FOXA1 function in breast cancer may be beneficial for patient outcomes. However, whether FOXA1 can serve as a therapeutic target in this disease is not yet clear. There are several lines of evidence that support pharmacological inhibition of FOXA1 in breast cancer. First, FOXA1 is a critical determinant of estrogen receptor signaling and is required for cell cycle progression and proliferation of luminal breast cancer, which accounts for the majority of breast cancer diagnoses [38,43,44,45,46,47,61]. FOXA1′s dysregulated expression and function, due to amplification, mutation, or upregulation, also drives resistance to endocrine and HER2-targeted therapies leading to worse patient outcomes [55,88,91,93,131]. Third, NR crosstalk can reprogram FOXA1 chromatin binding, which can also contribute to therapy resistance and compensatory pathway activation [58,148,149,150,154,156,158,168,169]. Increased FOXA1 expression can also suppress the tumor immune response, which is associated with worse patient outcomes [162,167,170]. Lastly, genetic approaches inhibiting FOXA1 expression within the contexts mentioned above result in decreased tumor cell proliferation, reversal of therapy resistance, and increased the tumor immune response. Together these data support inhibiting FOXA1 for breast cancer management. Despite this evidence, the development of small molecular inhibitors targeting transcription factors like FOXA1 remains challenging, as this class of proteins lacks ligand binding pockets for inhibitor docking. The advent of PROTACS (proteolysis targeting chimeras) has shown promise in degrading difficult to target molecules with several such chimeras already in clinical trials [171]. It is important to note that PROTACS are large complexes and their ability to traverse the blood brain barrier, a common metastatic site in TNBC, can be challenging [172]. Alternatively, discovering “druggable” factors that are required for FOXA1 expression, or identifying key downstream factors that mediate the effects of FOXA1 in endocrine resistance phenotypes may also be of clinical benefit. 

In this regard, Cocce and colleagues identified the GPI-anchored membrane protein, LYPD3, as a “druggable” downstream target of FOXA1 and the transcription factor, GRHL2 (Grainyhead Like Transcription Factor 2) in endocrine-resistant breast cancer [173]. Together, FOXA1 and GRHL2 induce the expression and activity of LYPD3, a Ly6 receptor family member, as well as its ligand AGR2, in mouse models of endocrine resistant breast cancer, as well as patient tumors that progressed on endocrine therapy. Inhibition of LYPD3 activity using a monoclonal antibody, reversed disease course in xenografted cell line models, providing feasibility for identifying targetable pathways downstream of FOXA1 that may be of therapeutic value. While thwarting FOXA1 expression may provide utility in breast cancer, it is also important to note that loss of FOXA1 can induce more primitive cell behaviors as discussed above [67]. Thus, preclinical assessment of a variety of models and diverse phenotypes will be necessary to ensure that such inhibitors do not induce more aggressive disease.

## 14. Conclusions

Over the past two decades, FOXA1 has been shown to play diverse roles in organ development as well as in several diseases, including breast cancer. In this context, FOXA1 is a pioneering factor for multiple nuclear hormone receptors and is associated with favorable patient outcomes, particularly in ER expressing luminal breast cancers, which represent the majority of cases. However, in other subsets of breast cancer, including TNBC and endocrine-therapy resistant tumors, FOXA1 is associated with worse clinical outcomes. Moreover, FOXA1 suppresses the tumor immune response independent of its licensing function, rendering tumors insensitive to immune checkpoint blockade. Thus, FOXA1 has been proposed to be a useful therapeutic target for treating aggressive breast lesions. However, the complexity of the roles of FOXA1 and its lack of binding pockets for small molecule docking will make it challenging to target. Thus, discerning the mechanisms that regulate FOXA1 expression and precisely identifying the tumor types that would respond favorably to FOXA1 inhibition will be essential and require more sophisticated model systems reflecting the complex hormonal milieu. Lastly, there are likely to be many additional roles of FOXA1 that will be revealed over the next two decades, including those independent of steroid nuclear receptor function. An increased understanding of the role of FOXA1 in both transcriptional induction and repression could inform improved treatment selection for patients with the potential of extending patient lives.
